# An unusual cause of recurrent urinary tract infection in a male patient and diagnostic challenge: a case report

**DOI:** 10.1016/j.ijscr.2025.111543

**Published:** 2025-06-19

**Authors:** Said Darwesh, Hilary Chipongo

**Affiliations:** a Department of Urology, Shree Hindu Mandal Hospital, P.O BOX 581, Dar es Salaam, Tanzania; b Critical care department, Shree Hindu Mandal Hospital P.O BOX 581, Dar es Salaam, Tanzania

**Keywords:** Bladder stones, Recurrent urinary tract infections

## Abstract

**Introduction:**

Approximately 5 % of all urological stones are attributed to bladder stones. Several risk factors are associated with the development of bladder stones including urinary stasis. Conditions such as benign prostate hypertrophy may cause bladder stones to develop in males. This is a unique cause of urinary tract infection without prostate enlargement resulting in a giant bladder stone in male patient.

**Case presentation:**

A 42-year-old male patient presented with complaints of on and off lower abdominal pains for more than 2 months associated with frequent urination. He has been treated multiple times for urinary tract infection without success.

**Discussion:**

Urinary tract infection in male patients, especially adults is a rare entity. This is a case of recurrent UTI resulting from two giant bladder stones in resource-limited settings.

**Conclusion:**

Urinary tract infections in males should not be overlooked. Hidden predisposing factors such as bladder stones may result in this entity. Proper imaging modalities including plain radiographs should be used as the initial management plan for males presenting with features suggestive of urinary tract infection.

## Highlights

• This is a case of recurrent UTI in an adult male patient treated without success.
• This article highlights the importance of using more than one diagnostic criteria in male patients thought to have lower urinary tract symptoms.

## 1 Introduction

Bladder stones constitute approximately 5 % of all urinary tract stones, and stones larger than 4 cm in diameter are considered rare [1]. The etiology of bladder stones is vast and the common predisposing factors which are known to date from the literature are, urinary stasis, neurogenic bladder disorder, urinary tract infections and foreign body [1,2]. Recurrent infection can be one of the notable causes of bladder stones which may be overlooked in males. There is a two-way relationship between bladder stones and the risk of developing urinary tract infections and vice versa as reported in the literature [2,3]. We report a case of complicated cystitis resulting from a huge bladder stone missed by normal conventional ultrasonography.

This case report has been reported in line with the SCARE Criteria [8].

## 2 Case presentation

**Fig. 1:**
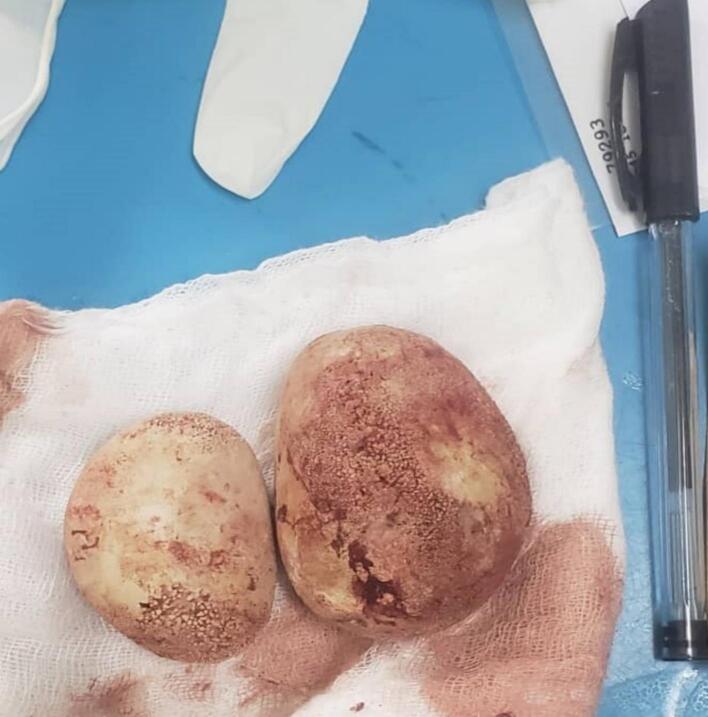
Showing two giant bladder stones extracted from a patient after performing a cystostomy due to failure of conventional cystoscopy.

A 42-year-old male patient presents in the outpatient clinic with a complaint of lower abdominal pain for the past 2 months. This condition is on and off for the entire period associated with increased urinary frequency and episodes of nausea. He has been treated in periphery health centres with a diagnosis of urinary tract infections where he was prescribed several antibiotics without any success. Renal function test was normal, random blood glucose was 4.7 mmol and he had no history of any other chronic illness. Urinalysis done showed leukocyte+++ in urine, KUB-ultrasound showed features of chronic cystitis and other structures were uneventful. The patient was planned for elective cystoscopy which initially failed thus open cystostomy was done and the findings were two large stones shown in Fig. 1.

## 3 Follow up

After surgery, the patient voided per SPC (supra-pubic catheter) to enable healing and was uneventfully followed for 8 weeks in the urology clinic. No complications resulted from the procedure, and the patient had normal renal functions.

## 4 Discussion

Literature shows that bladder stones are less common and usually weigh less than 100 g [1]. This pathology accounts for about 5 % of all urinary tract infections and 1.5 % of all admitted urological cases [1]. In endemic areas, it results from poor nutritional and socio-economic status where it mostly affects children [2]. In sub-Saharan countries, bladder stones are expected to be more prevalent in children compared to the adult population. In this case, we present a male patient of old age who had a history of recurrent urinary tract infections as a result of two giant bladder stones for more than 3-months.

The presentation usually mimics features of irritative and obstructive lower urinary tract symptoms which may include dysuria, recurrent urinary tract infections, urinary retention and in complicated cases haematuria [3]. Literature shows that they are usually single but in some rare cases, multiple stones may be present up to 30 % of cases [3]. Our patient presented with a history of on-and-off lower abdominal pain for more than 2 months associated with recurrent urinary tract infections which were treated multiple times with antibiotics without success.

Bladder stones are usually diagnosed by clinical evaluation of the obstructive and irritative voiding symptoms which in males are confused with features of prostate enlargement [3]. Recurrent urinary tract infection is one of the notable risk factors for bladder stones, but sometimes chronic urinary tract infections may be a result of obstructive uropathy caused by bladder stones [4]. Ultrasound scans have been reported to have a sensitivity and specificity for detecting bladder stones between 20 and 83 % and 98–100 % respectively, in which all are operator-dependent [5] in which cases may be missed out sometimes. Hence there is importance in using a multimodal approach to diagnosis such as a combination of ultrasound and plain radiographs.

Giant bladder stones are surgically managed either by open surgery. Literature shows the best approach used for the removal of giant bladder stones to be cystostomy, this is due to the fact that other modalities such as transurethral lithotripsy cannot remove large stones and is associated with an increased risk of infection [6,7].

## 5 Conclusion

Urinary tract infections in males should not be overlooked. Hidden predisposing factors such as bladder stones, may result in this entity. Proper imaging modalities including plain radiographs should be used as the initial management plan for males presenting with features suggestive of urinary tract infection.

## Consent

Written informed consent was obtained from the patient for publication and any accompanying images. A copy of the written consent is available for review by the Editor-in-Chief of this journal on request.

## Ethical approval

Our institution does not require ethical approval for reporting individual cases or case series.

## Funding

This article was not funded.

## Author contribution

Said Darwesh: writing the original manuscript.

Hilary Chipongo: literature search.

## Guarantor

I Hilary Chipongo I am the guarantor of this article.

## Research registration number

Shree Hindu Mandal Hospital does not offer ethical approval for single case reports. Hence ethical approval for publication was not required as per institutional policy.

## Conflict of interest statement

None of the authors has any conflict of interest to disclose. This is following the journal's guidelines.
